# Single-Trial EEG-fMRI Reveals the Generation Process of the Mismatch Negativity

**DOI:** 10.3389/fnhum.2019.00168

**Published:** 2019-05-28

**Authors:** Qiang Li, Guangyuan Liu, Guangjie Yuan, Gaoyuan Wang, Zonghui Wu, Xingcong Zhao

**Affiliations:** ^1^Education Science College, Guizhou Normal College, Guiyang, China; ^2^College of Electronic and Information Engineering, Southwest University, Chongqing, China; ^3^Chongqing Collaborative Innovation Center for Brain Science, Southwest University, Chongqing, China; ^4^College of Music, Southwest University, Chongqing, China; ^5^Southwest University Hospital, Southwest University, Chongqing, China

**Keywords:** EEG, FMRI, MMN, generation process, single-trial EEG

## Abstract

Although research on the mismatch negativity (MMN) has been ongoing for 40 years, the generation process of the MMN remains largely unknown. In this study, we used a single-trial electro-encephalography (EEG)-functional magnetic resonance imaging (fMRI) coupling method which can analyze neural activity with both high temporal and high spatial resolution and thus assess the generation process of the MMN. We elicited the MMN with an auditory oddball paradigm while recording simultaneous EEG and fMRI. We divided the MMN into five equal-durational phases. Utilizing the single-trial variability of the MMN, we analyzed the neural generators of the five phases, thereby determining the spatiotemporal generation process of the MMN. We found two distinct bottom-up prediction error propagations: first from the auditory cortex to the motor areas and then from the auditory cortex to the inferior frontal gyrus (IFG). Our results support the regularity-violation hypothesis of MMN generation.

## Introduction

The mismatch negativity (MMN) is a change-specific component of the event-related brain potential (ERP). This component is elicited when a repeating stimulus (termed the standard stimulus) is occasionally exchanged by a different stimulus (termed the deviant stimulus). The MMN is a negative difference waveform with a frontocentral scalp distribution peaking between 100 and 200 ms. Studies of the MMN have been frequent since its discovery in 1978 by Näätänen et al. (1978). Over 2,000 publications on the MMN have been reported (Bendixen et al., 2012). The reason why the MMN has been the subject of copious research is that it has been linked to two important concepts of experimental psychology: memory and attention (Näätänen et al., 1987; Näätänen and Winkler, 1999; Walker et al., 2001; Pincze et al., 2002; Winkler, 2007).

Three interpretations of the generation of MMN have been offered. The first is the hypothesis of refractory effects (Näätänen, 1990, 1992). This hypothesis suggests that the N1 amplitude is sensitive to refractoriness or adaptation (Budd et al., 1998; May et al., 1999) and thus the rare deviant stimuli elicit an enhanced N1 wave compared with the refracted N1 response elicited by the frequently presented standard stimuli. The second is the memory-mismatch explanation (Näätänen, 1990, 1992; Näätänen and Winkler, 1999). This hypothesis argues that MMN is a separate ERP component which reflects the difference between the deviant and the memory trace of the standard stimulus. This hypothesis is widely accepted and gives the MMN its name. The third theory of MMN generation is the regularity-violation hypothesis. Winkler (2007) summarized this hypothesis and provided a list of points tending to refute the memory-mismatch explanation (Horváth et al., 2001; Paavilainen et al., 2001, 2007). The regularity-violation hypothesis suggests that the repetitive aspects of standard stimuli form regularity representations in a predictive model in the brain. These representations encode rules extracted from regular inter-stimulus relationships. A new stimulus is compared with the regularity representations. The MMN reflects an updating process of representations whose prediction was mismatched to the most recent stimulus (Winkler, 2007).

According to the predictive-coding model of perception (Rao and Ballard, 1999; Friston, 2005, 2009; Arnal and Giraud, 2012), perception entails two distinct information propagations, the top-down propagation of prediction and the bottom-up propagation of prediction error (Hsu et al., 2015). These top-down and bottom-up propagations take place between hierarchical levels of the predictive model. The top-down propagation expresses prediction while the bottom-up propagation is a contingent error signal that updates the model (Rao and Ballard, 1999; Friston, 2005). Thus, verifying that the MMN is a sign of the propagation of a bottom-up prediction error can provide strong evidence for the regularity-violation hypothesis. However, until now the necessary direct observations have been lacking. One important reason for this situation has been the limitations of the available neuroimaging tools. Backward information streams flowing between hierarchical cortical areas are transient neural activities, and accessing these streams requires an imaging tool with both high temporal and high spatial resolution.

Neuroimaging tools can be divided into invasive and noninvasive methods. Invasive methods can assess neural activity with both high temporal and high spatial resolution. However, carrying out invasive studies on humans is challenging. Non-invasive methods such as functional magnetic resonance imaging (fMRI), electro-encephalography (EEG) and magneto-encephalography (MEG) also have limitations (please see reviews of Jorge et al., 2014; Murta et al., 2015). fMRI has high spatial resolution (at the millimetre level), but its low temporal resolution limits its ability to image transient neural activity (Eichele et al., 2005; Goldman et al., 2009; Walz et al., 2014; Iannaccone et al., 2015). EEG and its magnetic counterpart, MEG, have high temporal resolution (at the millisecond level) while their spatial resolution is low due to the inverse problem (Kalogianni et al., 2018): that is, the problem of extracting 3D source activity within the brain from 2D scalp recordings is ill-posed because different source configurations can generate the same electric potentials on the scalp (Helmholtz, 1853; Kalogianni et al., 2018).

A coupling of EEG-fMRI may generate a new method with both high spatial and high temporal resolution. Eichele et al. (2005) proposed an EEG-fMRI fusion method with the advantages of both earlier methods. fMRI exploits blood oxygenation level dependent (BOLD) response to assess intracranial neural activities. Logothetis et al. (2001) and Logothetis (2002) found that the BOLD response is approximately linearly related to the local field potential (LFP). The LFP is also the basis of the scalp EEG and the transient ERP (Buzsáki et al., 2012). Thus, the LFP is a hub that connects the BOLD response and the scalp EEG. The EEG-fMRI fusion method of Eichele et al. (2005) assumes linear coupling relationships among the BOLD, the LFP, and the ERP. This method utilizes the trial-to-trial variability of the ERP amplitude to anatomically locate local generators in the BOLD responses. This method can assess neural activity with both high temporal and high spatial resolution and has been widely used in studies of transient neuroimaging (Mulert et al., 2008; Jaspers-Fayer et al., 2012; Hoppstädter et al., 2015; Iannaccone et al., 2015).

In the present study, we use an auditory oddball paradigm to trigger an auditory MMN. We segment the MMN into five phases of 33.6 ms each (92–260 ms total) and trace the neural generators of these five phases using the method of Eichele et al. (2005). We find that the neural generators of the MMN comprise two distinct bottom-up propagations of neural activity. The first leads from the auditory cortex to motor areas and the second leads from the auditory cortex to the inferior frontal gyrus (IFG). In addition, our results reveal the time course of these propagations, and thus the detailed spatiotemporal generation process of the MMN. Our results support the regularity-violation hypothesis of the generation mechanism of the MMN (Winkler, 2007).

## Materials and Methods

### Participants

Thirty-two student participants (age 19–23; 15 males; all healthy and right-handed) were recruited at Southwest university. All participants were inquired before the experiment to make sure all of them are physically and mentally healthy. All participant hearing thresholds were tested and all were better than 25 dB from 250 Hz to 8,000 Hz. All participants were paid 120 RMB (about 18 dollars) for their participation and signed an informed consent. This experiment was approved by the ethics committee of Southwest University.

### Experimental Paradigm

The experimental paradigm of Hsu et al. (2015), which has been proven to elicit the MMN, was used in this study. Some changes were necessitated by the aims of the present study.

**Figure 1:**
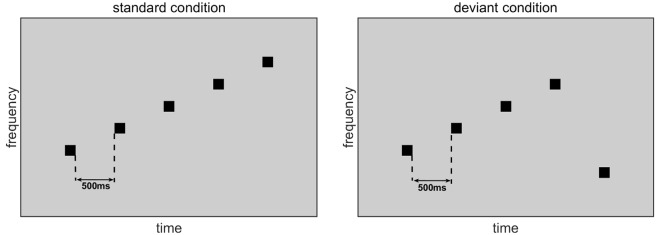
Standard-condition and deviant-condition stimuli. In the standard condition, five tones are arranged in ascending order by frequency. In the deviant condition, the first four tones are arranged in ascending order by frequency, but the fifth tone is one natural key lower than the first tone.

The sound stimuli consisted of seven pure tones. Each tone lasted 50 ms with 5-ms rising and falling edges. The frequencies of these tones were those of the seven natural keys of a modern piano, namely 261.626 (C4), 293.665 (D4), 329.628 (E4), 349.228 (F4), 391.995 (G4), 440.000 (A4), and 493.883 (B4) Hz. These tones were created by MATLAB 2015b. Figure 1 illustrates the stimuli used in the experiment. Each stimulus was a group of five tones chosen from the seven natural-key tones. In the standard condition, the five tones were arranged in order of ascending frequency in one natural key. In the deviant condition, which induces the MMN, the frequencies of the first four tones were identical to those of the standard stimulus, but the fifth tone was one natural key lower than the first tone. The ratio of the standard condition was six out of seven and the ratio of the deviant condition was one out of seven. In a group of five tones, the interval between tones was 500 ms. Standard and deviant conditions were randomly presented, with an interstimulus interval of 1.5–2 s.

An experiment comprised three runs. Each run comprised 24 trials with the deviant condition and 24 × 6 trials with the standard condition. The interval between runs were 1–2 min. Stimuli were presented using E-Prime 2.0, which was programmed to avoid the presentation of two deviant conditions in succession. Moreover, the first group presented was always the standard condition. During each run, the participants were instructed to watch a silent video film preselected by themselves and to pay no attention to the auditory stimulation. This served to prevent the elicitation of attention-dependent ERP components (Näätänen, 1982; Ritter et al., 1983; Sams et al., 1985; Shtyrov et al., 2003). At the onset time of a stimulus group, E-Prime 2.0 triggered the EEG-recording computer to record the onset times and categories of the stimuli.

To avoid interferences from scan noise, we used an active noise-reduction headphone (OptoACTIVE^1^), which was designed to remove 95% of scan noise. This headphone requires 16 s at the beginning of the experiment to learn the scan noise. Loudness of tones were normalized to 75 dB using a Bruel and Kjaer 2236 sound meter.

### Simultaneous Acquisition of EEG and fMRI

We acquired EEG and fMRI simultaneously in this study. EEG was recorded using a 32 electrodes MRI-compatible EEG recording system (BrainAmp MR plus, Brain products, Munich, Germany). Electrodes were placed according to the 10/20 system. An electrode was placed on the back to record the electrocardiogram. The sample rate of EEG recording was 5,000 Hz and all electrode impedances were below 10 kΩ.

Data was acquired at the brain imaging center of Southwest University using a 3-T Siemens MRI scanner with the following parameters: slice number = 32, voxel size = 3 × 3 × 3 mm, repetition time (TR) = 2 s, echo time = 26 ms, and flip angle = 90°. Each run comprised 350 scans. The first 10 scans were used to train the headphone, during which no stimuli were presented. After acquisition of the fMRI data, high-resolution, T1-weighted images were acquired with the following parameters: resolution = 1 × 1 × 1 mm, slice number = 176, TR = 1,900 ms, and echo time = 2.52 ms.

### EEG Preprocess and Analysis

Three kinds of noise were removed from the EEG, namely: (1) fMRI gradient artifacts; (2) ballistocardiogram (BCG) artifacts; and (3) ocular artifacts.

The first process was implemented in Analyzer 2.1 (Brain products, Munich, Germany). The EEG was first re-referenced to the TP9/TP10 electrode, then the fMRI gradient artifacts were removed using sliding average template subtraction (Allen et al., 2000) with Analyzer 2.1 and a cutoff frequency of 70 Hz. Then the EEG was down sampled to 500 Hz and exported to MATLAB for removal of the BCG artifacts.

The second step used MATLAB 2015b and the toolbox EEGLAB 13. The toolbox FMRIB 2.0 (Niazy et al., 2005) was used to remove the BCG artifacts using optimal basis sets. The parameter “number of PCs to use” was set at 3 (Niazy et al., 2005). Then the EEG was exported to Analyzer 2.1 for removal of ocular artifacts.

The Removal of ocular artifacts from the EEG was implemented in Analyzer 2.1 using the integrated function “Ocular remove using ICA.” Epochs were extracted from −100 to 300 ms from the onset of the fifth tone. Baseline was set to the −100–0 ms. We offered a picture of single-trial ERPs to the fifth tone of the standard condition as Supplementary Material (Figure S1), with −100 to 0 ms as the base line. It can be seen that after aforementioned preprocess, the signal-to-noise ratio of single-trial ERP has been acceptable.

Trials containing artifacts were automatically detected with a ±100 μV criterion. Visual inspection for residual artifacts was then performed (Jaspers-Fayer et al., 2012). Contaminated trials were discarded from the ERP analyses. The maximal number of discarded trials for a single participant was 4. However, in the EEG-fMRI analyses, contaminated trials were replaced by the average ERP for that condition. The criteria for detecting contaminated trials were the same as in the ERP analyses. Two participant’s data were discarded because of excessive residual artifacts. The following analyses, including ERP, fMRI, and EEG-fMRI, were implemented with the data of the remaining thirty participants. The single-trial ERP to standard tone of a subject was shown in the Supplementary Material as an example of signal-noise-ratio of single-trial ERP.

In this study, we analyzed ERPs from a pooled electrode comprised of FC1, FC2, Fz, and Cz, all located in a frontocentral area. Frontocentral areas have repeatedly proven to be the areas of greatest MMN amplitude; see reviews by Näätänen et al. (2007) and Bendixen et al. (2012).

### fMRI Preprocess

The fMRI data were pre-processed following the recommendations of the SPM12 Manual^2^. The preprocess procedure includes five steps: (1) head motion correction; (2) slice timing correction; (3) co-registration to anatomic structure to link the fMRI data to anatomic data of the T1-weighted image; (4) spatial normalization; and (5) gaussian smooth.

### fMRI and EEG-fMRI Analysis

Eichele et al. (2005) have proposed a single-trial EEG-fMRI analysis method that can take advantage of the high temporal resolution of EEG and the high temporal resolution of fMRI. Imaging a situation in which the brain responds to a stimulus sequentially in brain area A (100 ms) and B (200 ms) and generates an ERP at the mean time. Because the inner brain state inevitably fluctuates over time, the activation intensity of A and B fluctuates across trials. The Method of Eichele et al. (2005) assumes a linear relationship between single-trial ERP amplitudes and the activation intensities of the neural sources triggering the hemeodynamic response and then looks for brain regions in the fMRI data having the same pattern of fluctuations across trials as does an hemeodynamic response function (HRF)-convolved version of the single-trial ERP.

The activations in A and B can be depicted in two dimensions, onset time and amplitude. Assume that the onset times of A and B activations are 100 and 200 ms later, respectively, than the onset times of the stimuli. According to the linear assumption, the activation amplitudes of A and B across trials can be represented by the single-trial ERP amplitudes at 100 ms and 200 ms. Moreover, due to the low-pass characteristics of the HRF, the onset times of A and B can be simplified as the onset times of the stimuli. Thus, areas containing A and B can be located on the fMRI by regressors which contain the single-trial ERP-amplitude-modulated onset times of the stimuli. We implemented EEG-fMRI analysis using this method.

**Figure 2:**
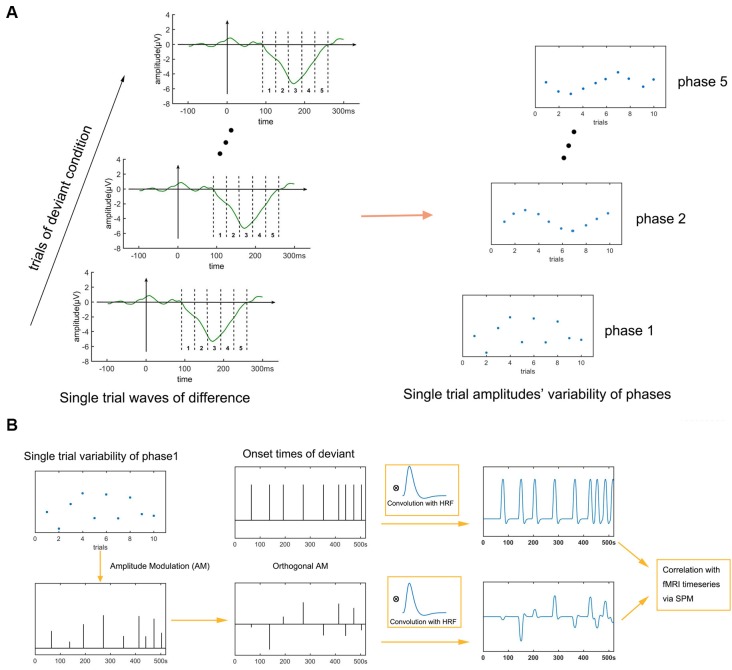
Flowchart of electro encephalography (EEG)-functional magnetic resonance imaging (fMRI) analysis. Two traditional regressors (the onset times of the standard and deviant conditions) and five phases of single-trial mismatch negativity (MMN) variation are entered into a general linear model (GLM) analysis of the fMRI data. Regressing the phases one at a time in sequence for each fMRI voxel and then identifying significant clusters of activated voxels reveals the spatiotemporal activation sequence of the MMN in the fMRI data. **(A)** Extraction of single-trial variability. **(B)** Fusion analysis.

fMRI and EEG-fMRI analyses were implemented after the preprocess of EEG and fMRI. The processing flowchart is shown in Figure 2. To summarize, seven regressors entered the first level general linear model (GLM) analysis (fixed effects analyses). Two regressors were calculated by convolving the onset times of the fifth tone in a group in the standard and deviant conditions with a canonical HRF. These two regressors represent obligatory responses to stimuli of constant amplitude.

In this current article, the EEG-fMRI fusion method mainly involves three steps: (1) extracting five phases’ single-trial amplitudes of the MMN; (2) amplitude-modulate traditional regressor with these single-trial amplitudes; and (3) traditional regressors (representing traditional fMRI analysis) and amplitude-modulated regressors (representing EEG-fMRI analysis) enter GLM analysis together. The detailed method follows. Single-trial difference waves were calculated by subtracting the ERP of the fifth tone of the previous standard condition from that of each deviant condition. To track the dynamic generating process of the MMN, we divided the MMN into five phases of 33.6 ms each (see Figure 2A) and analyzed the neural substrates of the five phases. The use of five phases was an empirical choice; too fine a division would lead to very similar regressors and overlapping activation results, whereas too coarse a division would lose detail unnecessarily. The beginning and end of the single-trial MMN were set as same as the mean MMN, that is from 92 ms to 260 ms. The amplitude of a phase of a trial was calculated by averaging the amplitude within the phase time at that trial. Thus five phases’ amplitudes across trials were extracted. The time series of the onset times of the deviant condition was amplitude modulated by the across-trial amplitudes of each of the five phases. These five amplitude-modulated time series were then decorrelated (Schmidt-Gram orthogonalization) with the onset times of the deviant condition to ensure that the activations associated to these time series were specific to the across-trial ERP fluctuation and not to stimuli presentation (Eichele et al., 2005; Jaspers-Fayer et al., 2012; Hoppstädter et al., 2015). These five amplitude modulated and decorrelated onset-time time series were convolved with the HRF to give five regressors capable of locating the neural sources of the five phases (Eichele et al., 2005; Walz et al., 2013; see also, review of Murta et al., 2015). Finally, a deviant vs. standard contrast and the neural sources of the five phases were analyzed with a GLM. Second level analyses (random effects analyses) were also performed with a significance of *P* < 0.05, cluster-extent family-wise error (FWE) corrected (voxel-height threshold *p* < 0.005).

## Results

### ERP Results

**Figure 3:**
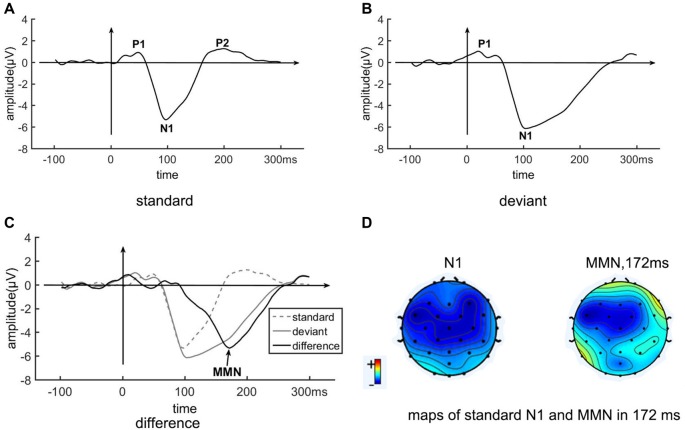
The event-related potential (ERP) of the standard stimulus, deviant stimulus, and MMN from the pooled electrode FC1/FC2/Fz/Cz. Panel **(A)** shown is the EPR of the standard condition. Panel **(B)** shown is the ERP of the deviant condition. Panel **(C)** shown is the EPR of the different wave (deviant—standard ERPs), as well as the EPR of the standard (dotted) and deviant (gray) conditions. **(D)** Maps of N1 of the standard condition and of the MMN at 172 ms, which is the peak time of the MMN.

Figure 3 shows the grand average ERP on the pooled electrode FC1/FC2/Fz/Cz. The ERP of the standard condition is a typical auditory ERP, with clear P1 (48 ms, 0.90 μV), N1 (98 ms, −5.34 μV) and P2 (200 ms, 1.24 μV) components. The ERP of the deviant condition is different from that of the standard condition, with an obviously slowly recovering N1 (104 ms, −6.15 μV). The difference between deviant and standard conditions shows an MMN covering about 92–260 ms, peaking at 172 ms, and reaching an amplitude of −5.34 μV. The ERP difference between standard and deviant conditions at 172 ms is significant, with *t* = 30.5, *p* < 0.0001. Both the N1 and the MMN are frontal-central distributed (Figure 3D).

### fMRI and EEG-fMRI Results

**Figure 4:**
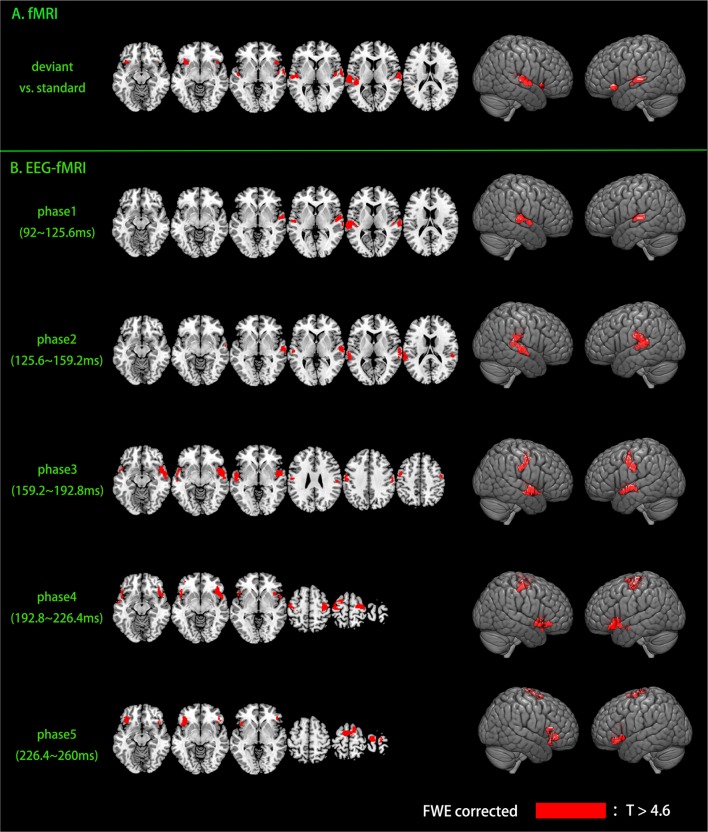
Activations of fMRI and EEG-fMRI. Activations were family-wise error (FWE) corrected (*p* < 0.05, voxel-height threshold *p* < 0.005). **(A)** fMRI results. **(B)** EEG-fMRI results.

**Figure 5:**
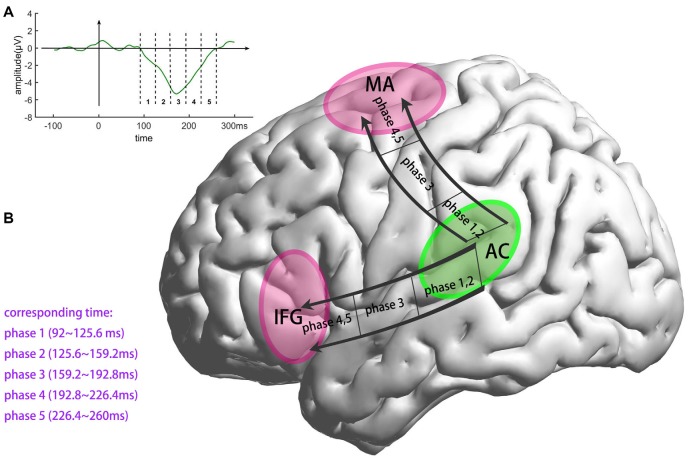
Illustration of the two distinct bottom-up, prediction-error propagations, both starting in the auditory cortex. The first advances to the motor areas. The second advances to the IFG along a different pathway. AC, the auditory cortex; MA, the motor areas; IFG, inferior frontal gyrus. **(A)** Division of MMN. **(B)** Generation process of MMN.

Traditional fMRI results are shown in Figure 4A and Table 1, results are reported with cluster size >10 voxels. Bilateral auditory cortex and bilateral inferior insula are found to be activated for the contrast of deviant vs. standard. Single-trial analysis results show the activations of the five phases of the MMN (shown in Figure 4B and Table 1, cluster size >10 voxels). The activations of phase 1 (92–125.6 ms) and phase 2 (125.6–159.2 ms) are in bilateral auditory cortex. The activations of phase 2 show a tendency to spread to areas outside the auditory cortex. The activations of phase 3 (159.2–192.8 ms) are in the bilateral superior temporal gyrus and the bilateral precentral gyrus. These activations are located between the phase 1, 2 and phase 4, 5. In phase 4 and 5 (192.8–260 ms), the activations are in the motor areas and the bilateral IFG. It can be seen that neural bases of these five phases form two bottom-up propagations (Figure 5). One is from the auditory cortex to the motor areas and the other is from the auditory cortex to the IFG.

**Table 1:** Functional magnetic resonance imaging (fMRI) and electro-encephalography (EEG)-fMRI results.

				MNI coordinates		
Condition	Peak Region	Cluster size	T	*x*	*y*	*z*
Deviant Vs. Standard	Left STG	531	6.72	−61	−19	6
	Right STG	274	6.37	63	−5	3
	Left Insula	170	6.57	−38	17	−9
	Right Insula	93	5.99	44	18	−5
Phase 1	Right STG	366	6.17	67	−21	7
	Left STG	329	5.85	−63	−19	8
Phase 2	Right STG	599	5.70	63	−17	1
	Left STG	656	6.01	−62	−32	13
Phase 3	Right STG	611	5.59	54	−3	−7
	Left STG	352	5.51	−56	−4	5
	Left Precentral Area	382	5.43	−48	−6	51
	Right Precentral Area	237	5.25	50	−12	50
Phase 4	Right Precentral Area	599	6.20	36	−18	64
	Left Precentral Area	576	5.98	−38	−19	68
	Right Insula	437	5.97	36	18	−10
	Left Insula	225	5.59	−40	12	−8
Phase 5	SMA	754	6.41	−8	−4	70
	Left IFG	364	5.41	−38	32	−10
	Right IFG	160	5.59	40	17	11
	Right IFG	106	5.20	45	35	−3

## Discussion

In this study, we used single-trial EEG-fMRI coupling to trace the generation process of the MMN. We divided the generation process of the MMN into five phases and resolved it into two bottom-up propagations from the auditory cortex, to motor areas and to the IFG, respectively. Our results are consistent with the theory of Winkler (Winkler, 2007) that the MMN is generated during the updating of a predictive model, which means that the propagations of the bottom-up prediction errors elicit the MMN.

### Traditional Results

In our results, the EEG response to the standard condition is a typical auditory ERP, with P1, N1 and P2 components. The ERP to the deviant condition shows a slowly recovering N1, resulting in the disappearance of the P2. The difference between deviant and standard conditions is a typical MMN, which begins at 92 ms and lasts to 260 ms. Our ERP results are consistent with previous MMN results (see reviews of Näätänen et al., 2007; Bendixen et al., 2012).

It has generally been thought that the auditory MMN is generated in the auditory cortex. For example, equivalent-current dipole studies have shown that the auditory cortex is the generator source of the MMN (Giard et al., 1995; Jemel et al., 2002; Hsu et al., 2015). Some MEG research has also indicated that the auditory cortex is the neural source of the MMN (Levänen et al., 1996; Alho et al., 1998; Hsu et al., 2014; Naeije et al., 2018). A near-infrared research confirmed that the auditory cortex generated the MMN (Rinne et al., 1999). Furthermore, invasive studies in animals (Ruusuvirta et al., 1998; Astikainen et al., 2000; Pincze et al., 2002; Harms et al., 2014) and humans (Rosburg et al., 2005; Dürschmid et al., 2016) have verified that the MMN is generated in the auditory cortex. However, some studies have also found neural sources of the MMN outside the auditory cortex. Using a lexical multiple-deviant oddball paradigm, Hsu et al. (2014) found the motor cortex and insula were activated when the deviant stimulus was presented. In a study in which the MMN was induced by duration and omission deviants, Recasens and Uhlhaas (Recasens and Uhlhaas, 2017) found that the motor cortex and some other cortices outside the auditory cortex were activated during the emergence of the MMN. In an invasive study using an auditory oddball paradigm, Phillips et al. (2016) found that the MMN was generated by the IFG and the temporal cortex. In a fMRI study, Molholm et al. (2005) found that the supratemporal and frontal cortices were activated when frequency and duration changes occurred. Our traditional fMRI results are consistent with these findings in that when the MMN occurs, the bilateral IFG are activated in addition to the auditory cortex. However, traditional fMRI results cannot reveal the generation process of the MMN.

### EEG-fMRI Results

Recent studies converge to suggest that the auditory cortex, the motor area, and the IFG form a predictive model during regular auditory perception. In a study of predictive processes in speech perception, Park et al. (2015) found causal top-down predictive signals in the IFG, including Brodmann area 44, 45 and 47 regions and the motor areas largely directed at the auditory cortex. They concluded that these top-down predictions improved processing of speech. Some speech comprehension studies (Davis and Johnsrude, 2007; Hervais-Adelman et al., 2012) have concluded that the auditory cortex is under the top-down influence of the IFG. The interpretations of the spoken input were found driven and constrained by top-down information flow from IFG. The speech prediction study of Cope et al. (2017) indicated that speech perception in the auditory cortex receives top-down prediction information from the frontal and motor areas. In a MEG study assessing temporal prediction, Morillon and Baillet (Morillon and Baillet, 2017) found that temporal prediction was generated in the motor areas and directed toward the auditory cortex. Moreover, Flinker et al. (2010) and Schneider et al. (2014) found that the auditory cortex is under top-down influence by the motor areas during vocalization. These studies indicate that during auditory perception, the auditory cortex, the motor areas and the IFG constitute a hierarchical predictive model. The motor areas and the IFG were identified as a higher level cortex sending top-down predictive information to the auditory cortex, identified as the lower level cortex.

In our experiment, the deviant stimuli violated stimulus regularity and therefore elicited the MMN. The paradigm used in this experiment avoided the induction of refractory effects because the frequently presented, standard sounds were not fixed, which would otherwise have produced a refracted N1 response. This serves to eliminate the refractory-effects hypothesis. According to the predictive coding theory, deviant stimuli should elicit propagation of bottom-up prediction errors. Consistent with this, our single-trial EEG-fMRI analysis showed that the neural bases of the five phases of the MMN formed two bottom-up propagations, one from the auditory cortex to motor areas, and the other from the auditory cortex to the IFG (see Figures 4, 5). In a former study regarding pure tone’s processing process, Li et al. (2019) found that when listen to a pure tone, the central nervous system process a pure tone in three phases: the first phase (~30 ms) in midbrain, the second phase (100–200 ms) in auditory cortex and the third phase (~400 ms) in motor cortex. In this article, although the fifth tone of deviant stimuli is still a pure tone, the processing process is largely different from the findings of Li et al. (2019). The regularity-violation hypothesis of MMN generation can well explain these results as the deviant stimuli causing prediction-model updating. The bottom-up propagations of prediction error would have elicited both the MMN, which can be recorded by EEG, and a hemodynamic response, which can be recorded by fMRI. By coupling the single-trial amplitudes of MMN and fMRI signals, the bottom-up, prediction-error propagations were retrieved. Our EEG-fMRI results revealed the detailed spatiotemporal generation process of the MMN and clearly supported the regularity-violation hypothesis.

It is worth to note that the paradigm used in this article to some extent is different from those used in the conventional MMN studies, which is mainly focused on automatic deviant detection. First, the number of auditory sequence is limited as there are only six possible sequences. Second, Figure 3B shows positive deflection prior to the onset in response to deviant tone. It may reflect some anticipating process. As the deviant tone comes only at the fifth tone in the five trains of tones, it might coincide with the predictive process focusing on the fifth tone. Taken together, the generation process analyzed in this article may not be comparable to the genuine MMN component.

Besides, in contrast to often used single-trial EEG-fMRI analysis method (Mulert et al., 2008; Iannaccone et al., 2015) which extracting components’ amplitude variability, our method used a less often used method (Jaspers-Fayer et al., 2012) that extracting single-trial amplitude variability according to fixed latency. The first method lay emphasis on the correlation between ERP components and BOLD signal, while the second method focuses on neural activity at fixed latency. As our aim was to detect the neural basis of different latencies of MMN, we used the second method in this study.

## Conclusion

Utilizing single-trial variability of the MMN, we analyzed the neuronal generators of the five phases of the MMN. We found two distinct propagation pathways for bottom-up prediction error, the first from the auditory cortex to the motor area, and the second from the auditory cortex to the IFG. This is the first assessment of the generating process of the MMN using non-invasive methods. Our results provide evidence supporting the regularity-violation hypothesis of the generator mechanism of the MMN.

## Data Availability

The datasets for this manuscript are not publicly available because due to the potential value that has not been fully explored by the current manuscript, survey respondents were assured raw data would remain confidential and would not be shared. Requests to access the datasets should be directed to 648047815@qq.com.

## Ethics Statement

This study was carried out in accordance with the recommendations of ethics committee of Southwest University with written informed consent from all subjects. All subjects gave written informed consent in accordance with the Declaration of Helsinki. The protocol was approved by the ethics committee of Southwest University.

## Author Contributions

QL designed the experiment, analyzed the data and wrote the article. GY did something in the experiment. GL designed the experiment. GW took part in the experiment and revised the article. ZW took part in the experiment and revised the article. XZ took part in the experiment and revised the article.

## Conflict of Interest Statement

The authors declare that the research was conducted in the absence of any commercial or financial relationships that could be construed as a potential conflict of interest.

## Footnotes

^1^http://www.optoacoustics.com/medical/optoactive-ii

^2^http://www.fil.ion.ucl.ac.uk/spm/

## Supplementary Material

FIGURE S1 An example of signal to noise ratio of single-trial ERP.
