# Supplementary figures and images for: Single-Trial EEG-fMRI Reveals the Generation Process of the Mismatch Negativity
Source: Front Hum Neurosci. 2019 May 28;13:168. doi: 10.3389/fnhum.2019.00168 (PMC6546813; doi:10.3389/fnhum.2019.00168)

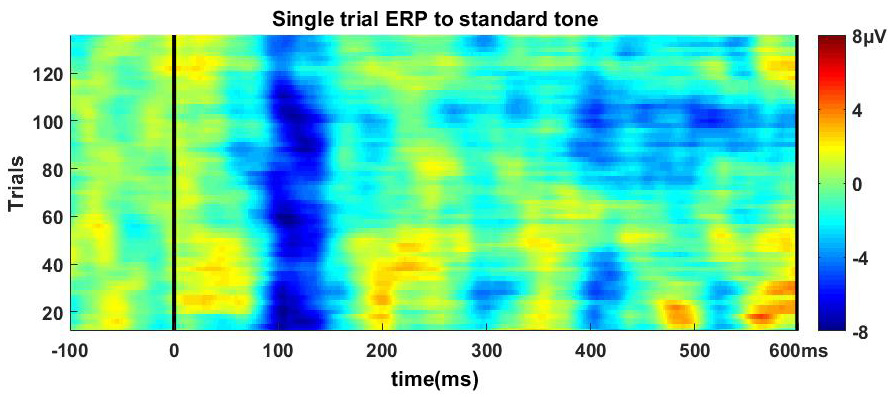

Supplement: FIGURE S1 — An example of signal to noise ratio of single-trial ERP. [file Image_1.JPEG]
